# Novel Association of High C-Reactive Protein Levels and A69S at Risk Alleles in Wet Age-Related Macular Degeneration Women

**DOI:** 10.3389/fimmu.2018.01862

**Published:** 2018-08-14

**Authors:** Patricia Fernandez-Robredo, Sergio Recalde, Maria Hernandez, Javier Zarranz-Ventura, Blanca Molins, Ricardo P. Casaroli-Marano, Alfredo Adan, Manuel Saenz-de-Viteri, Alfredo García-Layana

**Affiliations:** ^1^Experimental Ophthalmology Laboratory, Ophthalmology, Clínica Universidad de Navarra, Pamplona, Spain; ^2^Instituto de Investigación Sanitaria de Navarra (IdiSNA), Pamplona, Spain; ^3^Hospital Clínic, Institut Clínic d’Oftalmologia (ICOF), Barcelona, Spain; ^4^Fundació Clínic per a la Recerca Biomèdica, Institut d’Investigacions Biomèdiques August Pi i Sunyer (IDIBAPS), Barcelona, Spain; ^5^Ophthalmology, Clínica Universidad de Navarra, Pamplona, Spain; ^6^Royal Eye Infirmary, University Hospitals Plymouth NHS Trust, Plymouth, United Kingdom

**Keywords:** C-reactive protein, wet macular degeneration, polymorphism, single nucleotide, gender differences, case–control studies

## Abstract

**Purpose:**

To explore the relationship between plasma C-reactive protein (CRP) levels, the main *ARMS2* gene single nucleotide polymorphism (SNP), and gender in patients with neovascular age-related macular degeneration (wet AMD).

**Methods:**

Our study included 131 patients with wetAMD [age-related eye disease study (AREDS) category 4] and 153 control participants (AREDS category 1) from two Spanish retinal units. CRP levels were determined on blood samples by high-sensitivity ELISA assay. According to their CRP level, subjects were categorized into three well-established CRP categories: low (<1.00 mg/L, L-CRP), moderate (1–2.99 mg/L, M-CRP), and high (>3.00 mg/L, H-CRP). Genomic DNA was extracted from oral swabs using QIAcube (Qiagen, Hilden, Germany) and the A69S; rs10490924 of *ARMS2* gene was genotyped by allelic discrimination with validated TaqMan assays (Applied Biosystems, Foster City, CA, USA). Univariate and multivariate logistic regression adjusted for age was used to analyze the genomic frequencies and to calculate odds ratio (OR) using SNPStats software.

**Results:**

Considering CRP risk categories, H-CRP group showed a significant [OR 4.0 (1.9–8.3)] association with wetAMD compared to L-CRP group. The risk genotypes of A69S (TT) SNPs showed an association with wetAMD risk [OR 14.0 (4.8–40.8)]. Interestingly, the gender stratification of the CRP categories showed a significant increase in CRP levels in wetAMD women compared with control women [OR 6.9 (2.2–22.3)] and with wetAMD men [OR 4.6 (1.3–16.9)]. In addition, the subgroup analysis of CRP within A69S genotype and gender showed a link in women between the A69S and CRP levels in the AMD group compared to controls [OR 4.2 (1.4–12.6)].

**Conclusion:**

Our study shows, for the first time, that a different genetic association related with gender could contribute to AMD risk. As a consequence, the risk of female gender in the different CRP levels and A69S SNP frequencies could be taken into consideration to the established risk relationship of high levels of CRP and its association with risk A69S genotype.

## Introduction

Age-related macular degeneration (AMD) is the leading cause of irreversible vision loss among people more than 55 years of age in developed countries and can be divided into early and late stages. Late AMD is characterized by geographic atrophy or choroidal neovascularization (CNV) (wet AMD) both of which may lead to central visual impairment or irreversible blindness (1, 2). Inflammation and components of innate immunity, like complement activation, play a major role in the pathophysiology of AMD (3–5). Normal, adaptive parainflammation (defined as low-grade chronic inflammation) exists in the aging retina under physiologic conditions, but unbalanced and uncontrolled parainflammation leads to a detrimental chronic inflammatory response and contributes to the development of early and advanced AMD forms. This chronic process is mediated by many genetic and environmental factors (6).

Inflammatory factors, such as C-reactive protein (CRP), interleukin (IL)-6, and amyloid beta levels are increasingly being associated with AMD in the scientific literature (7–13). Among them, elevated CRP serum levels have been widely associated with both AMD development (9, 12, 14, 15) and progression (16). Similarly, several single nucleotide polymorphisms (SNP) in the complement pathway, particularly in the complement factor H (CFH), as well as in the *ARMS2* gene region, have been strongly associated with prevalence and incidence of AMD (17–19). More specifically, the A69S polymorphism has been related to the wet form of AMD rather than the atrophic form of the disease (20). Systemic CRP, along with genetic variants in the inflammatory pathway (CFH) and other pathway (ARMS2) have been associated with advanced AMD in age-related eye disease study (AREDS) sub-population (15). Although the relationship between CFH and CRP has been thoroughly studied by several authors, there is a paucity of high quality data directed to study the association between *ARMS2* and CRP levels.

Moreover, recent studies have shown controversial data about the influence of gender in the development and onset of early and late AMD. Whereas some studies have not found any relationship (1), a recent meta-analysis revealed that some individual studies suggested higher rates of neovascular AMD in women, mainly based on potentially higher rates of cerebrovascular events (2, 21). Interestingly, cerebrovascular and cardiovascular incidents have been related to increased systemic CRP levels (22).

To elucidate potential relationships between these factors, we aimed to investigate the influence of systemic CRP, A69S genotype, and gender in a large cohort of wetAMD patients and age-matched controls that attended in the Retinal units of two tertiary referral centers.

## Materials and Methods

### Study Population

Our study included 131 patients with wetAMD (AREDS category 4) and 153 control participants (AREDS category 1) from 2 tertiary referral hospitals: Clinica Universidad de Navarra and Hospital Clinic de Barcelona (from the Spanish Multicenter Group on AMD and the “Red Temática de Investigación Cooperativa en Salud,” RD07/0062, OFTARED RD12/0034 and RD16/0008). All participants were of Caucasian origin, completed a demographic questionnaire and gave permission for their inclusion in a database (data protection consent). All procedures were performed in accordance with the ethical standards of the Institutional Ethics Review Board of the Clínica Universidad de Navarra and with the 1964 Helsinki Declaration and its later amendments, or comparable ethical standards. All subjects gave written informed consent. Inclusion criteria for patients with wetAMD included the following: diagnosis of AMD with active subfoveal or juxtafoveolar CNV confirmed by fluorescein angiography (FA) and/or optical coherence tomography (OCT) (AREDS category 4). For control participants, inclusion criteria were the following: absence of drusen or no more than 5 small drusen (≤65 μm), absence of retinal pigment abnormalities in the macular area, and absence of chorioretinal macular atrophy or any other form of CNV (AREDS category 1). Exclusion criteria for this study (for both patients with wetAMD and control participants) included: age younger than 55 years, the presence of other CNV-related retinal diseases (i.e., angioid streaks, nevus in the macular area, toxoplasmosis scars, photocoagulation scars in the posterior pole, or polypoidal choroidal vasculopathy), history of retinal surgery, retinal disease in the studied eye (i.e., diabetic retinopathy or hereditary retinal dystrophies), and more than 6 diopters of myopia. DNA analysis was performed in 131 controls and 115 wetAMD subjects, the remaining samples were not analyzed because of technical problems with DNA (e.g., poor quality of DNA, DNA degradation, and sample collection issues). All cases underwent detailed ophthalmologic examination, including visual acuity assessment, dilated slit-lamp biomicroscopy, automatic objective refraction, color fundus photography, FA, and/or OCT. Controls underwent visual acuity assessment, mydriatic fundus examination, and measurement of refractive error and axial length.

### CRP Analysis

Samples were obtained from peripheral blood in EDTA containing tubes and processed accordingly (1,500 g, 15 min). Plasma high sensitive hsCRP levels were determined using a high-sensitivity ELISA assay (ICN Pharmaceuticals, Costa Mesa, CA, USA) as previously reported (23, 24) and following manufacturer instructions. All assays were performed in duplicates by investigators blinded to the clinical or genetic characteristics of the study subjects. Intra- and inter-assay coefficients of variations were <10%.

As previously reported by other authors (24) the distribution of CRP values was not normal (skewness = 1.130, kurtosis = 0.73), but the values transformed to the natural logarithm approximated a normal distribution (skewness = −0.846, kurtosis = 1.38). Logarithmic transformations were used for CRP for analyses with continuous variables in order to decrease the effect of extreme observations (25).

### Genotyping

The DNA samples from 131 controls and 115 wetAMD subjects were used in this study. Genomic DNA was extracted from oral swabs using QIAcube (Qiagen, Hilden, Germany). The control and wetAMD cohorts were genotyped for the rs10490924 of *ARMS2* gene by allelic discrimination with a validated assay (TaqMan; Applied Biosystems, Foster City, CA, USA) using real-time PCR (PE7300; Applied Biosystems), according to the manufacturer’s instructions.

### Statistical Analysis

Age analysis was performed by student’s *t*-test and chi-square test was used for gender distribution analysis. Levels of hsCRP and LogCRP were analyzed by lineal regression adjusted by age. The frequencies of alleles, genotypes, and haplotypes were calculated in all groups and were compared using a chi-square test and Fisher’s exact test, and corresponding ORs were calculated. A69S was in Hardy–Weinberg equilibrium.

Univariate logistic regression, adjusted by age and gender, and only by age for gender-based analysis, was used to estimate the ORs and 95% confidence intervals (95% CI) using SNPStats software (26). Analyses for each genetic variant were performed independently of other variants using codominant, dominant, recessive, and/or overdominant genetic models in base Akaike information, which chooses the inheritance model that best fits the data. A *p* value <0.05 was considered statistically significant.

## Results

### CRP Analysis

Demographics for gender distribution and mean age in control and wetAMD groups for the total population and the individuals that underwent A69S genotyping analysis are shown in Table 1. The mean age (years ± SEM) was 73.60 ± 0.57 for controls and 76.56 ± 0.61 for wetAMD group (*p* = 0.0005). The percentage of women was balanced in both controls and wetAMD patients (53.6 vs. 59.5%, respectively). No differences in age were observed between men and women both in control (73.30 ± 0.86 and 73.87 ± 0.77, respectively; *p* = 0.622) and wetAMD groups (76.83 ± 0.93 and 76.38 ± 0.80, respectively; *p* = 0.721). In men, the mean age was 73.30 ± 0.86 in controls and 76.83 ± 0.93 in wetAMD group (*p* = 0.007) and 73.87 ± 0.77 in control women vs. 76.38 ± 0.80 in wetAMD women (*p* = 0.025). Mean LogCRP levels, CRP distribution, and A69S genotypes according to wetAMD cases and controls are shown in Table 2. Regarding CRP systemic levels, significantly greater mean hsCRP (mg/L) levels normalized by Log function (24) were observed in wetAMD vs. controls (1.43 vs. 1.26, *p* < 0.001). Without normalization, these greater mean hsCRP levels were still statistically significant (3.47 vs. 2.71 mg/L, *p* < 0.001) and wetAMD individuals showed a 28% greater hsCRP level compared to controls. Usually, CRP levels are stratified into low (L-CRP, <1.0 mg/L), moderate (M-CRP, 1.0–3.0 mg/L), and high (H-CRP, 3.0–10.0 mg/L) (23, 24). In our study, we found that there was a statistical difference (*p* = 0.001) in CRP distribution between control and wetAMD participants. H-CRP levels were observed in 46.6% of wetAMD individuals compared to a 31.4% in controls, whereas L-CRP levels were observed in 9.9% of wetAMD group compared to 26.8% of controls. Compared to the L-CRP group, a greater wetAMD risk was observed for the M-CRP group (OR 2.8 95% CI, 1.3–5.8, *p* = 0.007) and H-CRP group (OR 4.0 95% CI, 1.9–8.3, *p* = 0.0001).

**Table 1:** Demographics for the total population and the subgroup which underwent A69S genotyping analysis.

		Control	WetAMD	*P* value (control vs. wetAMD*)*
**Total population**				
Men	*n* (%)	71 (46.4)	53 (40.5)	
	Age (years)	73.30 ± 0.86	76.83 ± 0.93	**0.007**
Women	*n* (%)	82 (53.6)	78 (59.5)	0.314
	Age (years)	73.87 ± 0.77	76.38 ± 0.80	**0.025**
Total	*n*	153	131	
	Age (years)	73.60 ± 0.57	76.56 ± 0.61	**0.0005**

**Subgroup DNA analysis**				
Men	*n* (%)	63 (48.1)	48 (41.7)	
	Age (years)	72.79 ± 0.89	77.10 ± 0.93	**0.001**
Women	*n* (%)	68 (51.9)	67 (58.3)	0.369
	Age (years)	73.87 ± 0.89	76.36 ± 0.91	0.052
Total	*n*	131	115	
	Age (years)	73.35 ± 0.63	76.67 ± 0.66	**0.0003**

*Age is shown in years (mean ± SEM). No significant differences were observed in gender distribution for the total population (*p* = 0.314) and the genotyped participants (*p* = 0.369). Comparisons in age within the total population between control men and women (*p* = 0.622), wetAMD men and women (*p* = 0.721) were not statistically significant. Comparisons within the genotyped population between control men and women (*p* = 0.395), wetAMD men and women (*p* = 0.577) were not statistically significant. Age analysis was performed by student’s *t*-test and chi-square was used for gender distribution. Significant p-values were highlighted in bold*.

**Table 2:** C-reactive protein (CRP) levels, number of subjects per CRP stratification, and A69S genotype for control and wetAMD groups.

	Control	WetAMD	*P* value	OR (95%CI)
Mean LogCRP ± SEM	1.26 ± 0.04	1.43 ± 0.03	**0.001**	

CRP—*n* (%)			**0.001**	
L-CRP (<1 mg/L)	41 (26.8)	13 (9.9)		1.00
M-CRP (1.0–3.0 mg/L)	64 (41.8)	57 (43.5)	**0.007**	**2.8 (1.3–5.8)**
H-CRP (3.0–10.0 mg/L)	48 (31.4)	61 (46.6)	**0.0001**	**4.0 (1.9–8.3)**

*ARMS2* A69S—*n* (%)	*n* = 131	*n* = 115	**<0.0001**	
GG	85 (64.9)	36 (31.3)		1.00
GT	41 (31.3)	54 (47.0)	**9.0 × 10^−5^**	**3.7 (2.0–6.8)**
TT	5 (3.8)	25 (21.7)	**1.0 × 10^−7^**	**14.0 (4.8–40.8)**
MAF	T: 0.19	T: 0.45	**7.6 × 10^−10^**	**3.4 (2.3–5.1)**

Mean LogCRP between controls and wetAMD participants was statistically significant (*p* < 0.001) based on lineal regression test and data on single nucleotide polymorphisms and CRP levels were analyzed by univariate logistic regression adjusted by age and gender. *L-CRP (low C-reactive protein, <1 mg/L), M-CRP (moderate C-reactive protein, 1.0–3.0 mg/L), and H-CRP (high C-reactive protein, 3.0–10.0 mg/L). SEM; OR, odds ratio; CI, confidence interval; MAF, minor allele frequency. Letters T and G: nucleotides for the *ARMS2* A69S. ORs >1 imply that was associated with increased risk of wetAMD when compared with the control (reference) group. Significant p-values were highlighted in bold.

### *ARMS2* A69S Genotyping

The distribution of genotypes for the A69S polymorphism was in Hardy–Weinberg equilibrium in the study population. The distribution of *ARMS2* A69S genotypes was different between controls and wetAMD cases (*p* < 0.0001, Table 2), as we have described previously (20). The study of allelic frequencies showed significant differences between wetAMD and control groups (OR 3.4 CI: 2.3–5.1; *p* = 7.6 × 10^−10^, Table 2). The percentage of risk genotypes (A69S GT and TT) was greater in the wetAMD group compared to the control group (68.7 vs. 35.1%). GT genotype showed a strong association with risk of wetAMD (OR 3.7 CI: 2.0–6.8; *p* = 9.0 × 10^−5^) which was even greater for the TT-risk genotype, with a 14-fold increase in wetAMD group vs. controls (OR 14.0 CI: 4.8–40.8; *p* = 1.0 × 10^−7^, Table 2).

### CRP and *ARMS2* A69S Genotypes and Gender Influence

#### CRP Analysis by Gender

Descriptive statistics divided by gender, control and wetAMD groups, CRP levels, genotype frequencies, and demographic factors are disclosed in Tables 3–6. Significantly higher LogCRP and hsCRP levels were observed in women between control and wetAMD groups (*p* = 0.002 and *p* = 0.009, respectively, Table 3), while men did not show differences between these groups (*p* = 0.140 and *p* = 0.275, respectively, Table 3). Women showed greater wetAMD risk in M-CRP [OR 5.7 (1.8–18.7); *p* = 0.002] and H-CRP groups [OR 6.9 (2.2–22.3); *p* = 0.0004] compared to the L-CRP group (Table 3). The percentage of patients with H-CRP levels was greater in wetAMD vs. controls in women [52.6 vs. 37.8%, OR = 6.9 (2.2–22.3), *p* = 0.0004], but no differences were observed in men [37.7 vs. 23.9%, OR = 2.6 (0.9–7.2), *p* = 0.08] (Table 3).

**Table 3:** Subgroup analysis by gender.

	Men				Women			

	Controls	WetAMD	*P* value	OR (95% CI)	Controls	wetAMD	*P* value	OR (95% CI)
Number per group—*n*	71	53			82	78		
Mean hsCRP (mg/L) ± SEM	2.63 ± 0.31	3.13 ± 0.32	0.275		2.79 ± 0.24	3.71 ± 0.25	**0.009**	
Mean LogCRP ± SEM	1.21 ± 0.06	1.36 ± 0.05	0.140		1.30 ± 0.04	1.49 ± 0.03	**0.002**	

C-reactive protein (CRP)—*n* (%)								
L-CRP	20 (28.2)	9 (17.0)		1.00	21 (25.6)	4 (5.1)		1.00
M-CRP	34 (47.9)	24 (45.3)	0.5	1.6 (0.6–5.0)	30 (36.6)	33 (42.3)	**0.002**	**5.7 (1.8–18.7)**
H-CRP	17 (23.9)	20 (37.7)	0.08	2.6 (0.9–7.2)	31 (37.8)	41 (52.6)	**0.0004**	**6.9 (2.2–22.3)**

*ARMS2* A69S—*n* (%)	*n* = 63	*n* = 48			*n* = 68	*n* = 67		
GG	40 (63.5)	21 (43.8)		1.00	45 (66.2)	15 (22.4)		1.00
GT	21 (33.3)	17 (35.4)	0.39	1.5 (0.6–3.51)	20 (29.4)	37 (55.2)	**1.2 × 10^−5^**	**8.8 (3.7–21.4)**
TT	2 (3.2)	10 (20.8)	**0.003**	**9.7 (1.9–50.1)**	3 (4.4)	15 (22.4)	**8.3 × 10^−6^**	**22.8 (5.4–95.2)**
MAF	*T*; 0.20	*T*; 0.39	**0.002**	**2.5 (1.4–4.6)**	*T*; 0.19	*T*; 0.50	**1.1 × 10^−7^**	**4.2 (2.4–7.3)**

*Mean C-reactive protein (CRP) level and number of men and women divided into control and wetAMD groups according to CRP groups and genotype frequencies*.

*L-CRP (low C-reactive protein, <1 mg/L), M-CRP (moderate C-reactive protein, 1.0–3.0 mg/L), and H-CRP (high C-reactive protein, 3.0–10.0 mg/L). LogCRP levels were analyzed by lineal regression and adjusted by age. CRP groups and A69S genotyping were analyzed by univariate logistic regression adjusted by age. SEM; OR, odds ratio; CI, confidence interval; MAF, minor allele frequency. Letters T and G: nucleotides for the *ARMS2* A69S. ORs >1 imply that was associated with increased risk of wetAMD when compared with the control (reference) group. Significant p-values were highlighted in bold*.

#### *ARMS2* A69S Genotypes Analysis by Gender

Subgroup analysis by A69S genotypes and gender revealed a risk association for TT genotype and wetAMD in men [OR 9.7 (1.9–50.1); *p* = 0.003, Table 3]. However, this association was observed in women for both the GT [OR 8.8 (3.7–21.4); *p* = 1.2 × 10^−5^] and TT [OR 22.8 (5.4–95.2); *p* = 8.3 × 10^−6^, Table 3] genotypes when comparing control and wetAMD groups. The allelic frequency studies also corroborated the genotyping results, and differences between the control group vs. wetAMD were observed in men [OR 2.5 (1.4–4.6), *p* = 0.002] and women, who showed a greater association [OR 4.2 (2.4–7.3), *p* = 1.1 × 10^−7^; Table 3].

**Figure 1:**
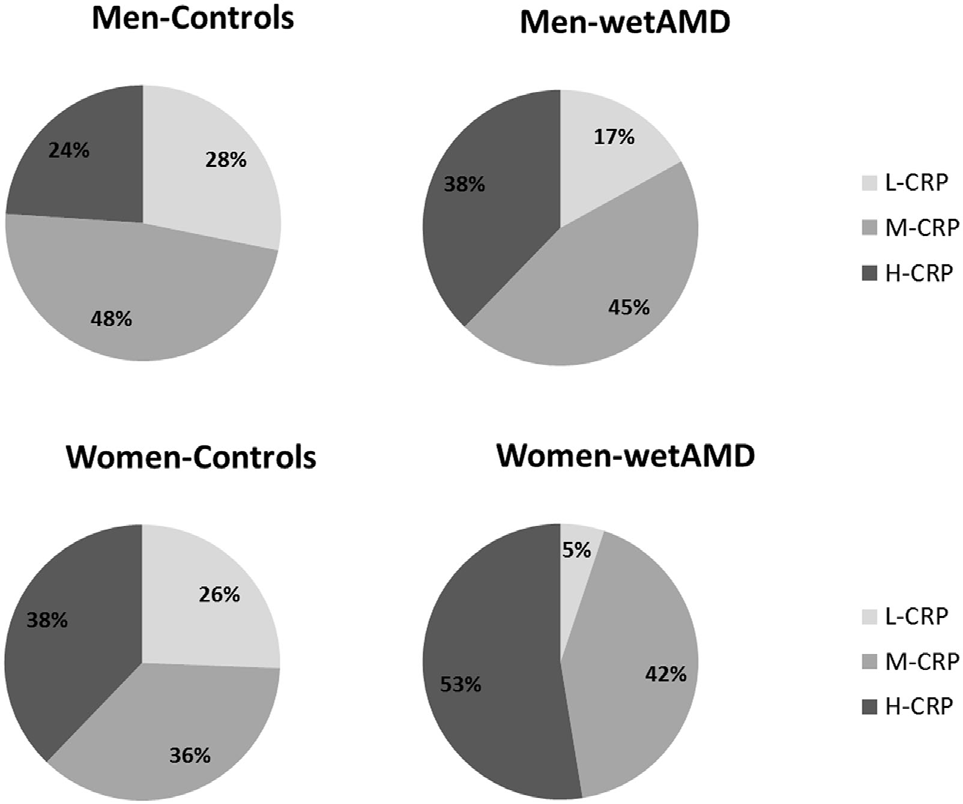
Percentage of individuals according to C-reactive protein (CRP) level stratification divided by gender (men: upper row; women: lower row) and study groups (controls: left column and wet age-related macular degeneration: right column). L-CRP (low C-reactive protein, <1 mg/L; light gray), M-CRP (moderate C-reactive protein, 1.0–3.0 mg/L; medium gray), and H-CRP (high C-reactive protein, 3.0–10.0 mg/L; dark gray). Letters T and G: nucleotides for the *ARMS2* A69S.

The results obtained from the division of control and wetAMD individuals into men and women are shown in Table 4. The analysis of the LogCRP levels showed that wetAMD women had significantly higher CRP levels compared with wetAMD men (1.49 vs. 1.36, *p* = 0.036, Table 4). Further, no differences were reported in the control group with regards to CRP stratification levels between women and men (Table 4; Figure 1). However, women with H-CRP levels showed higher risk of wetAMD compared to men [52.6 vs. 37.7%, OR 4.6 (1.3–16.9); *p* = 0.02]. No differences were observed for M-CRP or L-CRP groups, and interestingly, L-CRP levels were observed only in 5.1% of wetAMD women compared to 17% of wetAMD men (Table 4; Figure 1).

**Table 4:** Subgroup analysis for control and wetAMD individuals divided into gender, according to CRP groups and genotype frequencies.

	Control				WetAMD			

	Men	Women	*P* value	OR (95% CI)	Men	Women	*P* value	OR (95% CI)
Number per group—*n*	71	82			53	78		
Mean hsCRP (mg/L) ± SEM	2.63 ± 0.31	2.79 ± 0.24	0.696		3.13 ± 0.32	3.71 ± 0.25	0.154	
Mean LogCRP ± SEM	1.21 ± 0.06	1.30 ± 0.04	0.427		1.36 ± 0.05	1.49 ± 0.03	**0.036**	

C-reactive protein (CRP)—*n* (%)								
L-CRP	20 (28.2)	21 (25.6)		1.00	9 (17.0)	4 (5.1)		1.00
M-CRP	34 (47.9)	30 (36.6)	0.86	1.2 (0.5–2.6)	24 (45.3)	33 (42.3)	0.12	3.1 (0.8–11.2)
H-CRP	17 (23.9)	31 (37.8)	0.28	1.7 (0.7–4.0)	20 (37.7)	41 (52.6)	**0.02**	**4.6 (1.3–16.9)**

*ARMS2* A69S—*n* (%)	*n* = 63	*n* = 68			*n* = 48	*n* = 67		
GG	40 (63.5)	45 (66.2)		1.00	21 (43.8)	15 (22.4)		1.00
GT	21 (33.3)	20 (29.4)	0.70	0.9 (0.4–1.8)	17 (35.4)	37 (55.2)	**0.016**	**3.1 (1.3–7.6)**
TT	2 (3.2)	3 (4.4)	1.00	1.3 (0.2–8.3)	10 (20.8)	15 (22.4)	0.19	2.2 (0.8–6.2)
MAF	*T*; 0.20	*T*; 0.19	1.00	0.9 (0.5–1.7)	*T*; 0.39	*T*; 0.50	0.10	1.6 (0.9–2.7)

*L-CRP (low C-reactive protein, <1 mg/L), M-CRP (moderate C-reactive protein, 1.0–3.0 mg/L), and H-CRP (high C-reactive protein, 3.0–10.0 mg/L). LogCRP levels were analyzed by lineal regression and adjusted by age. CRP groups and A69S genotyping were analyzed by univariate logistic regression adjusted by age. SEM; OR, odds ratio; CI, confidence interval; MAF, minor allele frequency. Letters T and G: nucleotides for the *ARMS2* A69S. ORs >1 imply that was associated with increased risk of wetAMD when compared with the control (reference) group. Significant p-values were highlighted in bold*.

**Figure 2:**
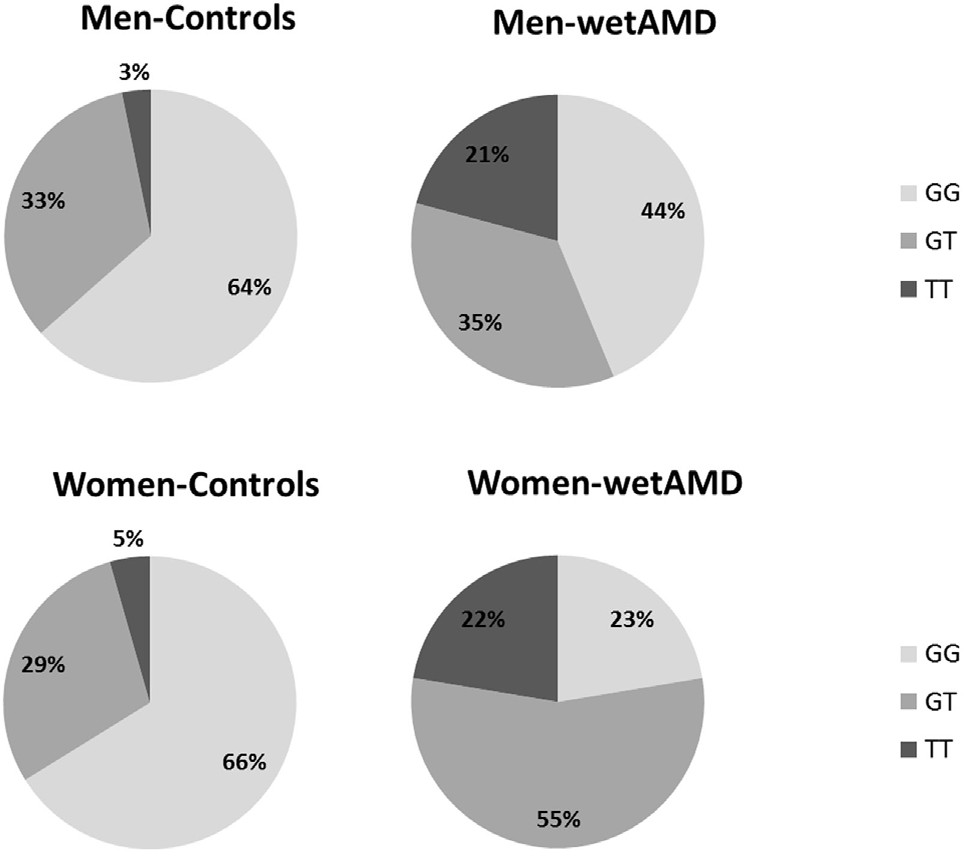
Percentage of individuals according to A69S genotypes divided by gender (men: upper row and women: lower row) and study groups (controls: left column and wetAMD: right column). L-CRP (low C-reactive protein, <1 mg/L; light gray), M-CRP (moderate C-reactive protein, 1.0–3.0 mg/L; medium gray), and H-CRP (high C-reactive protein, 3.0–10.0 mg/L; dark gray). Letters T and G: nucleotides for the *ARMS2* A69S.

With regards to A69S genotypes and gender of the same group, differences between wetAMD men and women were only observed in GT genotype frequencies [OR 3.1 (1.3–7.6); *p* = 0.016, Table 4; Figure 2]. The allelic study did not show significant differences for the T risk allele between wetAMD women and men, although the allelic frequency in women was higher than in men [*T*; 0.39 vs. 0.50, *p* = 0.10; OR 1.6 (0.98–2.7), Table 4; Figure 2].

#### *ARMS2* A69S Genotypes and CRP Combined Analysis by Gender

**Figure 3:**
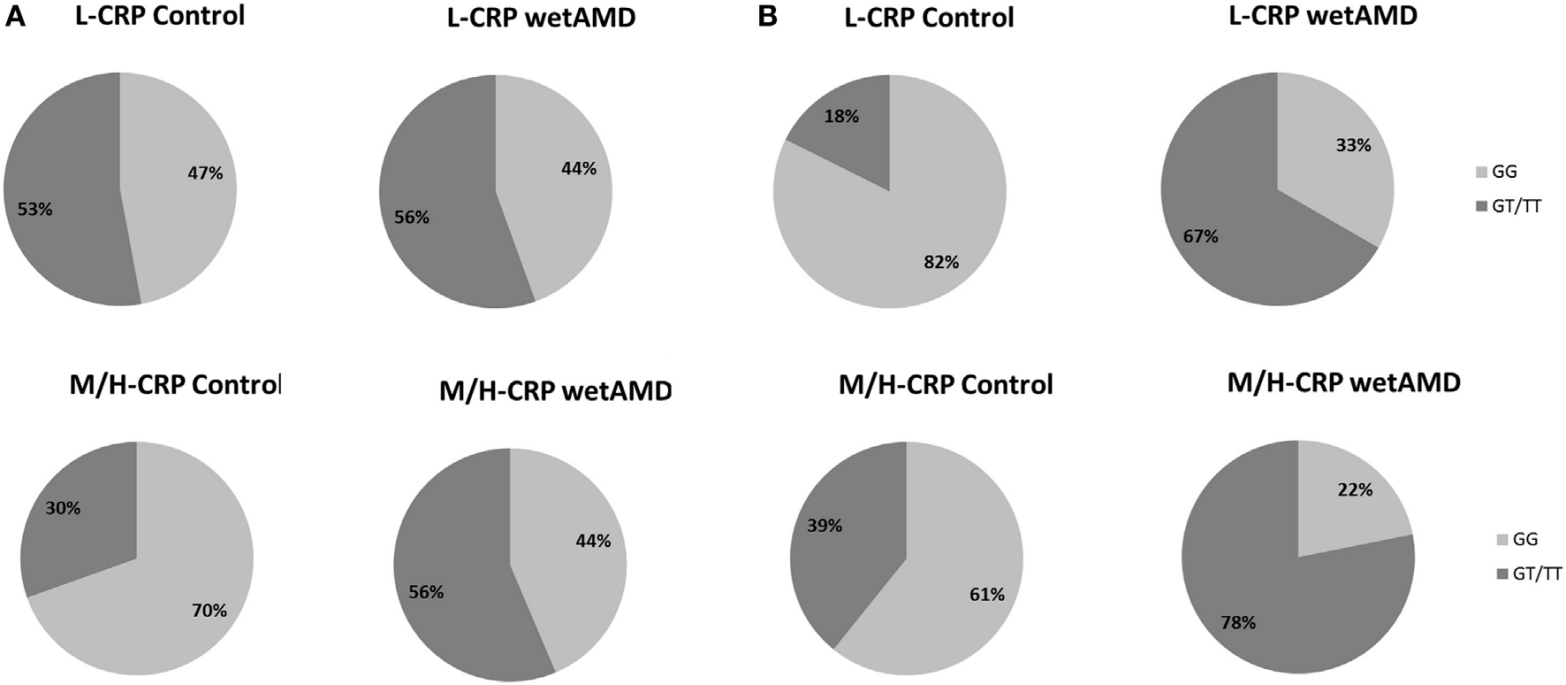
Percentage of individuals according to men **(A)** and women **(B)** and to C-reactive protein (CRP) stratified groups (L-CRP: upper row and M/H-CRP: lower row) and A69S genotype (light gray: GG and dark gray: GT/TT). L-CRP (low C-reactive protein, <1 mg/L) and M/H-CRP (moderate-high C-reactive protein, 1.0–10.0 mg/L). Letters T and G: nucleotides for the *ARMS2* A69S.

The distribution of A69S genotypes and stratified CRP levels divided by gender in both control and wetAMD patients is shown in Tables 5 and 6, respectively. These tables also show *p*-values of the interaction between CRP levels and A69S, based on a model controlling for dominant genotypes (GT/TT vs. GG) and CRP (L-CRP and M/H-CRP) adjusted by age. Normal population usually shows L-CRP levels and taking into account that a CRP level higher than 1 mg/L is considered a cardiovascular risk factor. Therefore, we combined both categories (M-CRP and H-CRP) into a single one (M/H-CRP) to assess the influence of CRP levels on the risk of wetAMD, depending on genotypes. A very strong relationship was observed between A69S risk genotypes, CRP levels, and female gender. In the total study cohort, the allele frequency study showed a significant association of the T risk allele in both CRP levels analyzed [L-CRP; OR = 3.2 (1.1–8.9), *p* = 0.03, and M/H-CRP; OR = 3.3 (2.1–5.2), *p* = 5.3 × 10^−8^, Table 5]. In M/H-CRP level, women showed a greater association of T risk allele compared with men [OR 3.4 (1.9–6.1), *p* = 2.0 × 10^−5^ and OR 3.0 (1.5–6.0), *p* = 0.001, Table 5; Figure 3]. Considering the GT/TT risk genotypes this difference is even greater. The total population showed a 4.3-fold increase likelihood of wetAMD in GT/TT genotypes compared to GG [OR 4.3 (2.4–7.8), *p* = 6.5 × 10^−7^, Table 5], however, was women carrying GT/TT distribution where the risk of wetAMD vs. control was the greatest [women; OR 5.5 (2.4–12.5), *p* = 2.4 × 10^−5^ and men; OR 3.0 (1.2–7.2), *p* = 0.014, Table 5]. We further explored these differences and found that, women with M/H-CRP and GT/TT genotypes have a threefold greater risk of wetAMD than men with such conditions [OR 2.8 (1.2–6.6), *p* = 0.03, Table 6; Figure 3]. Finally, a trend for a protective association was in the control group for women carrying GG and L-CRP compared to men [OR 0.2 (0.04–1.0), *p* = 0.07, Table 6].

**Table 5:** Dominant model of the frequencies observed for the *ARMS2* A69S genotype within CRP groups dividing total population, men and women into control and wetAMD groups.

ARMS2-A69S	Total population				Men				Women			
	
	Control	WetAMD	*P* value	OR (95% CI)	Control	WetAMD	*P* value	OR (95% CI)	Control	WetAMD	*P* value	OR (95% CI)
**L-CRP**												
GG	22	5		1.00	8	4		1.00	14	1		1.00
GT/TT	12	7	0.19	2.6 (0.7–9.9)	9	5	0.95	1.1 (0.2–5.6)	3	2	0.14	9.3 (0.6–139.6)
MAF	*T*; 0.18	*T*; 0.42	**0.03**	**3.2 (1.1–8.9)**	*T*; 0.25	*T*; 0.39	0.35	1.9 (0.6–6.4)	*T*; 0.09	*T*; 0.50	**0.03**	**10.3 (1.4–75.7)**

**M/H-CRP**												
GG	63	31		1.00	32	17		1.00	31	14		1.00
GT/TT	34	72	**6.5 × 10^−7^**	**4.3 (2.4–7.8)**	14	22	**0.014**	**3.0 (1.2–7.2)**	20	50	**2.4 × 10^−5^**	**5.5 (2.4–12.5)**
MAF	*T*; 0.20	*T*; 0.46	**5.3 × 10^−8^**	**3.3 (2.1–5.2)**	*T*; 0.17	*T*; 0.38	**0.001**	**3.0 (1.5–6.0)**	*T*; 0.22	*T*; 0.50	**2.0 × 10^−5^**	**3.4 (1.9–6.1)**

*L-CRP (low C-reactive protein, <1 mg/L) and M/H-CRP (moderate-high C-reactive protein, 1.0–10.0 mg/L). Data were analyzed by univariate logistic regression adjusted by age and gender for the total population and by age for men and women subgroups. OR, odds ratio; CI, confidence interval; MAF, minor allele frequency. Letters T and G: nucleotides for the *ARMS2* A69S. ORs >1 imply that was associated with increased risk of wetAMD when compared with the control (reference) group. Significant p-values were highlighted in bold*.

**Table 6:** Dominant model of the frequencies observed for the *ARMS2* A69S genotype within CRP groups between control and wetAMD in men and women separately.

ARMS2-A69S	Control				WetAMD			

	Men	Women	*P* value	OR (95% CI)	Men	Women	*P* value	OR (95% CI)
**L-CRP**								
GG	8	14		1.00	4	1		1.00
GT/TT	9	3	0.07	0.2 (0.04–1.0)	5	2	1.0	1.6 (0.1–24.6)
MAF	*T*; 0.25	*T*; 0.09	0.10	0.3 (0.06–1.1)	*T*; 0.39	*T*; 0.50	0.9	1.5 (0.2–10.3)

**M/H-CRP**								
GG	32	31		1.00	17	14		1.00
GT/TT	14	20	0.4	0.7 (0.3–1.6)	22	50	**0.03**	**2.8 (1.2–6.6)**
MAF	*T*; 0.17	*T*; 0.22	0.5	0.7 (0.3–1.5)	*T*; 0.38	*T*; 0.50	0.11	1.6 (0.9–2.8)

*L-CRP (low C-reactive protein, <1 mg/L) and M/H-CRP (moderate-high C-reactive protein, 1.0–10.0 mg/L). Data were analyzed by univariate logistic regression adjusted by age. OR, odds ratio; CI, confidence interval; MAF, minor allele frequency. Letters T and G: nucleotides for the *ARMS2* A69S. ORs >1 imply that was associated with increased risk of wetAMD when compared with the control (reference) group. Significant p-values were highlighted in bold*.

## Discussion

The present study describes, for the first time, a novel association between gender, CRP (a systemic marker of inflammation), the A69S SNP in the *ARMS2* gene, and wet AMD. We found that CRP is a multiplying wet AMD risk factor to the *ARMS2* SNP in women, and we observed a threefold higher wetAMD risk for women carrying the GT or TT at-risk allele and with intermediate or high CRP levels compared to men.

Age-related macular degeneration is a chronic and complex disease affecting people older than 55 years (1) in which several genetic and environmental factors have an influence on its progression (27, 28). Although serum CRP levels have gained greater importance as a risk marker in cardiovascular disease (CVD), the magnitude of its risk potential has been debated (29). Consistent evidence of a significant association between elevated serum CRP and late AMD supports the hypothesis that higher levels of CRP (>3 mg/L) are associated with a twofold higher likelihood of late AMD. In contrast, the evidence of an association between CRP and early AMD is weaker (23, 30). This difference has been previously investigated for other diseases in which CRP is considered an inflammatory marker, such as CVD (31), obstructive sleep apnea (32), and obesity (33), with controversial results. In the present study, we confirmed our previous results where a relationship between A69S and the risk of wetAMD was observed (20). We have found a fourfold increase of wetAMD likelihood in GT and a 14-fold increase for the at-risk TT genotype. Since elevated CRP confers a higher risk of AMD (30), the combination of these findings suggests that the risk allele might contribute to the development of AMD. In our study, we observed that *ARMS2* A69S heterozygous and homozygous risk alleles along with moderate CRP levels seem to be associated with wet AMD in men and women.

ARMS2 protein is a component of the extracellular matrix of the choroid that some authors locate in the mitochondria (34). Recent studies in rats, using electron microscopy, have demonstrated disorganization and swelling of mitochondria’s after CRP injection in cardiomyocytes from ischemic carotids (35). Other authors have found that ARMS2 is located in the cytosol colocalized with cellular skeleton (microtubules and actin filaments), suggesting that A69S may gain function to interact with the cytoskeleton (36). Furthermore, CRP is involved in microtubules stabilization by inhibiting neutrophil movement through the kinases pathway (37). Finally, some authors have found significantly higher CRP deposits in the Bruch’s membrane and retinal and choroid vessels of wet AMD eyes compared to aged controls and AMD human donors (38).

Currently, there is great concern about the role of gender as potential bias that may influence results of clinical trials and affect diagnostic or treatment decisions. As a consequence, gender perspective has recently become a major factor to be considered in clinical research and especially in genetics (39), since some differences related to gender may commonly be missed and/or remain unrevealed. To ascertain these potential differences, we considered the parameters separately for each gender. After dividing the study cohort into men and women, we observed a strong association between high levels of CRP and wetAMD in women, which was not seen in men. Moreover, in the subanalysis performed by gender in control and wetAMD groups, higher levels of CRP were more frequently observed in women compared with men within the wetAMD group. These results could suggest an association of female gender and high CRP levels and allegedly, inflammation, in the development of wet AMD. These findings highlight the potential usefulness of systemic CRP as a marker of wet AMD in women, as a novel risk factor that may also be considered along with the well-established factors for AMD. Interestingly, wetAMD men only showed a 56.2% of heterozygous and at-risk homozygous genotypes compared to the 77.6% observed in wetAMD women. Furthermore, women with GT genotype had a threefold increase risk of wetAMD compared to men.

Understanding the combined effects and interactions among specific genotypes and risk factors in wet AMD may provide new insights into the complex etiology of such chronic condition. In fact, the study of the association between AMD genetics, inflammatory factors, and gender is a rapidly evolving research field. A recent study, for example, described that *DAPL1* is an AMD-associated gene and that this disease association is female-specific (40).

Despite the growing consensus that chronic inflammation is an important factor in the pathogenesis of AMD, few studies have studied the association of *ARMS2* and systemic inflammatory markers such as CRP. Hence, we also analyzed the correlation between serum CRP categories and *ARMS2* A69S genotypes in the total population and stratified by gender. In our study, in agreement with the results observed by Seddon et al. (15), we observe that the *ARMS2* at-risk allele for AMD is strongly associated with moderate and high CRP systemic levels with a fourfold increased risk, suggesting that probably this association is related to low-grade chronic systemic inflammation (22).

The most novel finding in our study is related to gender. We found that women with intermediate-high CRP and carrying the A69S at-risk alleles had almost a sixfold greater risk of wetAMD compared to the threefold risk observed in men. This was confirmed when genders were compared within groups, and an almost threefold higher risk of wetAMD was observed in women with the at-risk GT/TT alleles and moderate-high CRP levels compared to men.

We hypothesized that a plausible biological explanation to these gender-related differences reported could rely on the different hormone levels observed in men and women. Oral hormone therapy and CRP levels in postmenopausal women have shown a direct relationship (41). Further, a biological pathway for AMD that features hormone replacement therapy includes estrogen receptors 1 and 2. Both proteins have been observed in the human retina, suggesting that estrogens play a role in the pathogenesis of AMD (42, 43). Estrogen controls the expression of chitinase 3-like-1 protein (YKL-40), a molecule found in choroidal neovascular membranes. Lower levels of estrogen may trigger the upregulation of YKL-40 and play a role in the development of neovascular AMD (44). Moreover, estrogens have an antioxidant effect by inhibiting lipid peroxidation, which provides protection against oxidative damage in the retina caused by the aging process (45). Women are mostly exposed to hormonal changes with aging and especially after their mid-life, which may contribute to a greater risk compared to men who usually show more stable hormonal levels. However, the exact mechanisms underlying these differences are still unclear and these preliminary results come from preclinical investigations using cell culture and animal models.

This is the first study directed to evaluate the relationship between the *ARMS2* A69S distribution, serum CRP levels, and gender in AMD patients and controls. Interestingly, we have found a very strong association in women between the *ARMS2* A69S and CRP levels in the AMD group compared to controls. The mechanisms and associations related to the *ARMS2* locus and CRP remain to be elucidated. In our study, it seems that the *ARMS2* and intermediate-high CRP levels provide greater risk of wetAMD, especially in women.

However, it should be noted that these findings may be considered within the context of a selected population and some limitations should be acknowledged such as sample size. Therefore, more studies to replicate this association in an independent sample set in a stronger powered analysis are needed. Moreover, as other authors have also reported for CVD, CRP levels can be confounded by obesity, ethnicity, gender, and other comorbidities (31). Future studies must consider the inclusion of factors, such as smoking, body mass index, serum cholesterol, hypertension, and the use of hormone replacement therapy in the variance component model.

In spite of the limitations described above, our study shows several strengths including that this is the first time that a gender-based analysis on A69S genotype frequencies is performed. Despite the relatively low number of samples in the gender subgroups, the magnitude of the associations and the significance values obtained are strong enough to consider these aspects for future prospective and retrospective studies. These data can serve as a starting point for prospective trials evaluating such associations. Taking into account these results, a potential therapeutic approach could be directed to modify this systemic inflammation status, especially among those genetically high-risk individuals carrying the at-risk alleles in order to reduce the likelihood of wet AMD and, according to other studies, also other forms of late AMD (46).

In conclusion, we have observed that women with high CRP levels showed higher risk of wetAMD than men, suggesting that systemic CRP levels could be useful as an important multiplying factor to the already established genetic factors involved in the development of wet AMD. These individuals should be prioritized for interventional studies directed to prevent the progression of the disease (47). One proposal for those at-risk populations has been the inhibition of monomeric-CRP for local treatment of vascular disease (48) and reduction of myocardial infarct size (49) in at-risk areas. In line with this, monomeric-CRP has shown to induce an inflammatory phenotype (50) and blood–retinal barrier disruption in RPE cells (51). Given the similarities of risk factors between CVD and AMD, this approach could be considered as a local adjuvant therapy in future new strategies for patients at greater risk, such as women with high CRP levels and at-risk A69S genotype (52). This could contribute to a better quality of life and reduce personal burden for AMD patients.

## Ethics Statement

All procedures were performed in accordance with the ethical standards of the Institutional Ethics Review Board of the Clínica Universidad de Navarra and with the 1964 Helsinki Declaration and its later amendments, or comparable ethical standards. All subjects gave written informed consent.

## Author Contributions

Conceptualization: PF-R, AG-L, SR, and JZ-V. Data curation: PF-R, SR, and JZ-V. Funding acquisition: AG-L and PF-R. Investigation: JZ-V, RC-M, AA, SR, and AG-L. Methodology: BM, SR, MV, JZ-V, and PF-R. Resources: JZ-V, RC-M, AA, and AG-L. Supervision: AG-L, PF-R, and AA. Writing—original draft: PF-R, SR, and MH. Writing—review and editing: PF-R, MV, BM, MH, AG-L, SR, AA, and JZ-V.

## Conflict of Interest Statement

The authors declare that the research was conducted in the absence of any commercial or financial relationships that could be construed as a potential conflict of interest. The handling Editor is currently co-organizing a Research Topic with one of the authors BM, and confirms the absence of any other collaboration.
